# Identifying degenerative effects of repetitive head trauma with neuroimaging: a clinically-oriented review

**DOI:** 10.1186/s40478-021-01197-4

**Published:** 2021-05-22

**Authors:** Breton M. Asken, Gil D. Rabinovici

**Affiliations:** 1 Department of Neurology, Memory and Aging Center, Weill Institute for Neurosciences, University of California, San Francisco, 675 Nelson Rising Lane, Suite 190, San Francisco, CA 94143 USA; 2 Departments of Neurology, Radiology & Biomedical Imaging, Memory and Aging Center, Weill Institute for Neurosciences, University of California, San Francisco, 675 Nelson Rising Lane, Suite 190, San Francisco, CA 94143 USA

**Keywords:** Chronic traumatic encephalopathy, Traumatic encephalopathy syndrome, Traumatic brain injury, Repetitive head trauma, Concussion, Neuroimaging, Positron emission tomography, Magnetic resonance imaging, Neurodegenerative disease

## Abstract

**Background and Scope of Review:**

Varying severities and frequencies of head trauma may result in dynamic acute and chronic pathophysiologic responses in the brain. Heightened attention to long-term effects of head trauma, particularly repetitive head trauma, has sparked recent efforts to identify neuroimaging biomarkers of underlying disease processes. Imaging modalities like structural magnetic resonance imaging (MRI) and positron emission tomography (PET) are the most clinically applicable given their use in neurodegenerative disease diagnosis and differentiation. In recent years, researchers have targeted repetitive head trauma cohorts in hopes of identifying in vivo biomarkers for underlying biologic changes that might ultimately improve diagnosis of chronic traumatic encephalopathy (CTE) in living persons. These populations most often include collision sport athletes (e.g., American football, boxing) and military veterans with repetitive low-level blast exposure. We provide a clinically-oriented review of neuroimaging data from repetitive head trauma cohorts based on structural MRI, FDG-PET, Aβ-PET, and tau-PET. We supplement the review with two patient reports of neuropathology-confirmed, clinically impaired adults with prior repetitive head trauma who underwent structural MRI, FDG-PET, Aβ-PET, and tau-PET in addition to comprehensive clinical examinations before death.

**Review Conclusions:**

Group-level comparisons to controls without known head trauma have revealed inconsistent regional volume differences, with possible propensity for medial temporal, limbic, and subcortical (thalamus, corpus callosum) structures. Greater frequency and severity (i.e., length) of cavum septum pellucidum (CSP) is observed in repetitive head trauma cohorts compared to unexposed controls. It remains unclear whether CSP predicts a particular neurodegenerative process, but CSP presence should increase suspicion that clinical impairment is at least partly attributable to the individual’s head trauma exposure (regardless of underlying disease). PET imaging similarly has not revealed a prototypical metabolic or molecular pattern associated with repetitive head trauma or predictive of CTE based on the most widely studied radiotracers. Given the range of clinical syndromes and neurodegenerative pathologies observed in a subset of adults with prior repetitive head trauma, structural MRI and PET imaging may still be useful for differential diagnosis (e.g., assessing suspected Alzheimer’s disease).

**Supplementary Information:**

The online version contains supplementary material available at 10.1186/s40478-021-01197-4.

## Introduction

Lifetime head trauma exposure is a risk factor for multiple neurodegenerative diseases including Alzheimer’s disease (AD), Parkinson disease, amyotrophic lateral sclerosis, and chronic traumatic encephalopathy (CTE) [1–8]. CTE is the only neurodegenerative disease occurring almost exclusively in individuals with prior repetitive head trauma exposure, which is often sustained in the context of collision sports and/or military service. Conversely, most individuals with non-CTE neurodegenerative diseases have no *documented* head trauma exposure history, especially repetitive. Neuroimaging plays a key role in informing differential diagnoses of suspected neurodegenerative diseases, but researchers thus far have had little success identifying diagnostic imaging biomarkers that are specific for CTE in living adults.

Several advanced neuroimaging techniques have helped rapidly advance our understanding of disease pathophysiology, though only a handful are utilized clinically. Structural magnetic resonance imaging (MRI) is routinely performed in clinical settings to rule out non-neurodegenerative causes of clinical impairment like mass lesions and stroke, but also provides topographic representation of atrophy patterns. In some neurodegenerative conditions, atrophy patterns are sensitive and specific enough that they improve diagnostic certainty of syndromes like Alzheimer’s-type dementias [9–11], primary progressive aphasia subtypes [12], and behavioral variant frontotemporal dementia [13]. However, frank volume loss observable on structural MRI is thought to represent the culmination of a complex process of protein misfolding and deposition, neuronal and glial dysfunction, and ultimately synaptic and neuronal loss. Structural MRI interpretation also can be complicated by image processing limitations. Brain structure visualization and volume quantification via MRI occurs indirectly through measurement of underlying physical–chemical properties of brain tissue. There is inherent risk of overattributing group differences to true structural changes when other potential confounds like tissue perfusion or water content variability also significantly influence the measurement outcomes [14].

Other imaging techniques like positron emission tomography (PET) provide opportunities for detecting evidence of disease pathophysiology upstream of degeneration and potentially prior to symptom onset in individuals at risk for neurodegenerative disease. PET imaging is employed clinically to quantify metabolic brain changes or the burden and distribution of Aβ plaques and tau tangles using radiolabeled tracers. Uptake of fluorodeoxyglucose (FDG) is a marker of synaptic activity and brain metabolism. There is limited research applying FDG-PET in cases of repetitive head trauma or suspected CTE. Radiotracers have also been developed for binding Aβ plaques and tau tangles. Aβ tracers most often used in clinical practice include Florbetapir, Florbetaben, and Flutemetamol. Regarding tau PET, early small studies of repetitive head trauma cases used the FDDNP tracer, which has significant limitations, including nonselective binding to multiple insoluble protein aggregates (e.g., Aβ, tau, prion protein and others), low signal-to-noise ratio, and poor reproducibility across centers. Flortaucipir, the most studied tau PET tracer, was developed to bind paired helical filaments of tau that form neurofibrillary tangles (NFTs) in AD. AD research has rapidly facilitated development and clinical translation of Aβ and tau PET radiotracers. Together, these PET tracers can essentially confirm presence of AD plaque and tangle pathology in living patients. As we later discuss, initial excitement over the potential utility of tau PET tracers for identifying CTE tau pathology has significantly tempered in recent years. Other PET tracers detecting important disease processes like neuroinflammation (e.g., translocator protein, or TSPO-PET) [15] or synapse loss currently have relatively little data in repetitive head trauma populations.

The suspected higher prevalence of CTE in clinically impaired adults with repetitive head trauma exposure has motivated efforts targeting this population. Traumatic encephalopathy syndrome (TES) refers to the clinical manifestations of cognitive and/or neurobehavioral changes in individuals with repetitive head trauma exposure [16, 17]. Research criteria for TES were revised in 2021 [17] and require “substantial” exposure to repetitive head impacts from collision sports, military service, or other causes to qualify for diagnosis. There must be a predominant cognitive (episodic memory and/or executive functioning) and/or neurobehavioral syndrome (explosiveness, impulsivity, rage, etc.). Symptoms must be progressive and not fully accounted for by another neurologic, psychiatric, or medical condition, though suspicion of comorbid conditions (e.g., another neurodegenerative disease) is not exclusionary. A provisional level of certainty is assigned based on degree of head impact exposure and specific symptom manifestations - “Suggestive of CTE,” “Possible CTE,” or “Probable CTE”. Fluid and neuroimaging biomarkers do not factor into these research diagnostic criteria for TES due to lack of available data demonstrating specific associations with CTE pathology. “TES with definite CTE” can only be diagnosed by autopsy.

TES criteria were designed initially to maximize sensitivity over specificity to underlying CTE pathology. Earlier criteria [16] included several core and supportive symptoms also observed in relatively high frequency among adults without repetitive head trauma exposure or neurodegenerative disease (e.g., depression, anxiety, headaches) [18, 19]. Changes on structural neuroimaging (e.g., cavum septum pellucidum) or PET imaging of Aβ plaques and tau tangles previously were proposed to inform the likelihood that CTE is the cause of TES. However, neuroimaging correlates of repetitive head trauma, with or without presumed CTE, remain incompletely characterized. In particular, recent studies suggest that the proposed use of currently available tau PET tracers for increasing diagnostic certainty of CTE is premature.

The large number of review articles focused on neuroimaging of repetitive head trauma reflects the scientific interest in establishing neuroimaging biomarkers of CTE or other neurodegenerative effects of repetitive head trauma [20–29]. There are two key concepts that must be emphasized when reviewing and interpreting this literature: 1) clearly understanding the definition, frequency, severity, and timing of the head trauma exposure in any given study, and 2) resisting the temptation to assume that significant neuroimaging findings within repetitive head trauma cohorts reflect biomarkers specific to CTE. Despite CTE being highly associated with repetitive head trauma exposure [30, 31], CTE is only one possible neurodegenerative outcome of repetitive head trauma and often exists with other pathologies, such as Aβ plaques, alpha-synuclein, TDP-43 proteinopathies, and white matter rarefaction [5, 32]. In other words, the association of CTE with prior repetitive head trauma is much stronger than the association of repetitive head trauma with underlying CTE.

## Scope of the Review and Relevant Terminology

Here we provide an overview of structural MRI and PET neuroimaging data from *repetitive head trauma populations,* with a focus on literature published within approximately the past 5 years. We chose to highlight structural MRI and PET given their direct clinical applications. Data from advanced neuroimaging modalities like diffusion tensor imaging (DTI), functional MRI, cerebral perfusion, and other modalities advance our understanding of repetitive head trauma pathophysiology. However, these modalities currently have minimal clinical footprint and largely are not validated for informing differential diagnosis.

“Head trauma” represents a complicated clinical spectrum that spans asymptomatic to symptomatic trauma. Table 1 describes classifications of commonly used terms in head trauma research as applied in this review. We focused our overview on repetitive head trauma populations, which we defined by individuals exposed, at a minimum, to *repetitive, asymptomatic head impacts* (i.e., “subconcussive” trauma without observable or reported acute symptoms*)*. Typically, these populations are current or former collision sport athletes exposed to up to tens of thousands of asymptomatic head blows throughout a playing career, or military servicemembers subjected to repeated blast exposures. These groups inherently are at high risk of sustaining multiple *symptomatic* events, often mild TBI or concussion, in addition to repetitive asymptomatic blows. Other research has focused on acute and chronic neuroimaging outcomes in groups defined by discrete, symptomatic TBI events. Occasionally, such studies incorporate “repeat TBI” groups defined by having more than one symptomatic TBI. Such populations differ from those highlighted in our review based on the absence of repetitive, asymptomatic trauma, which currently is believed to be more strongly associated with TES and CTE.

**Table 1:** Head trauma terminology

Head trauma terminology	Definitions and typical contexts
Traumatic Brain Injury (TBI)	Symptomatic injury often requiring presence of *either* loss of consciousness (LOC) *or* posttraumatic amnesia (PTA) Crudely delineated as “mild,” “moderate,” or “severe” based on duration of LOC or PTA or using Glasgow Coma Scale score More commonly applied in studies of civilian populations in emergency department settings or military servicemembers and veterans than in sport-related head trauma settings
Concussion	Symptomatic injury often considered interchangeable with “mild TBI” In sport settings, concussion diagnoses frequently are made without documented LOC or PTA, but based on presence of other head trauma-associated symptoms like headache, dizziness, poor balance, nausea, or eye movement abnormalities, among others Instances of “getting your bell rung,” “seeing stars,” or “clearing the cobwebs” typically qualify for a concussion diagnosis
Subconcussive Trauma	Asymptomatic head impacts usually occurring in the context of collision sports In collision sports like American football, athletes may sustain tens of thousands of asymptomatic, subconcussive head impacts in the course of an extended playing career In military settings, servicemembers may experience subconcussive exposure in the form of repeated blast exposures or training activities (e.g., breacher or combat training) without associated acute clinical symptoms
Traumatic Encephalopathy Syndrome (TES)	Research criteria proposed for classifying cognitive and neurobehavioral *symptoms* believed to be associated with repetitive head trauma and with onset typically years after last head trauma exposure TES diagnostic criteria have high sensitivity but low specificity to underlying CTE neuropathology In this review, “TES” refers to study populations defined by clinical symptomatology in the context of prior repetitive head trauma, without presumption of underlying CTE neuropathology
Chronic Traumatic Encephalopathy (CTE)	*Neuropathologic changes* found in the brain per consensus diagnostic criteria (phosphorylated tau protein aggregates in neurons around blood vessels at the depths of cortical sulci) CTE diagnosis is made independent of patient symptoms during life In this review, “CTE” refers only to study populations with autopsy-confirmed evidence of CTE neuropathology

Several terms lack consensus and there remains controversy regarding optimal characterization. These definitions were applied in the manuscript but may not directly overlap with use in other brain injury settings

In most cases, the repetitive head trauma studies referenced throughout include individuals who fulfill the minimum exposure criterion proposed in the TES research diagnosis [17]. We reserve the use of *“TES”* for populations defined by clinical symptomatology in the context of prior repetitive head trauma exposure, without presumption of known underlying CTE pathology. *“CTE”* will be used when referring to neuropathologic changes found in the brain at autopsy per consensus diagnosis recommendations [33], without presumption of a specific clinical manifestation.

## General Challenges and Limitations of Current Research

Validating neuroimaging modalities as CTE biomarkers currently has significant challenges. Existing diagnostic criteria aimed at identifying living adults with underlying CTE are likely to capture many “false positive” patients given the criteria’s emphasis on sensitivity over specificity. Repetitive head trauma exposure places individuals at higher risk of CTE, but most will not develop CTE, so research cohorts defined by exposure alone may not have a high rate of CTE. Autopsy is currently the only gold standard for developing CTE biomarkers, and CTE cases with antemortem clinical and neuroimaging data are exceedingly rare [34–37]. There is also little research that has directly compared patients with and without prior head trauma with the cognitive and neurobehavioral features of the proposed TES criteria, which may help clarify syndrome profiles with greater specificity to head trauma-related neurodegenerative disease, like CTE.

Variability in acquisition of head trauma exposure data also complicates interpretation of current literature. Most neuroimaging studies of repetitive head trauma cohorts draw comparisons to either clinically normal or impaired controls considered free of lifetime head trauma exposure. Head trauma researchers frequently raise concerns about inaccurate characterization of exposure, though discussions usually focus on improving accuracy of exposure estimates in the head trauma cohorts themselves. Arguably, this issue is just as relevant for identifying appropriate control groups. Screening questionnaires inquiring about prior brain injury can be markedly insensitive and rarely query for lifetime participation in high-risk activities like collision sports [38]. Many questionnaires also require LOC or PTA for an event to qualify as a brain injury. There is therefore a high likelihood that many “control” groups used in these studies include some individuals with exposure to milder head trauma (e.g., concussion without LOC or PTA) or repetitive asymptomatic impacts, especially if drawn from existing study cohorts that were not recruited explicitly to serve as unexposed controls in comparison to a repetitive head trauma group. Inclusion of comparison groups with participants that have prior head trauma exposure may reduce the likelihood of identifying significant differences in various neuroimaging outcomes. However, matching controls on non-head trauma variables also presents challenges because high risk groups like elite athletes may disproportionately include individuals with sociodemographic, personality factors (e.g., risk-taking behaviors), and cognitive strengths (e.g., visuospatial or processing speed abilities) that are not representative of the general population.

## Structural MRI

### Brain Volume Differences Associated with Repetitive Head Trauma

Head trauma can result in diffuse axonal injury (DAI) resulting from the shear-strain forces imparted on white matter tracts [39]. Severe forms of TBI can result in DAI, focal contusions, or hemorrhages observable on conventional clinical MRI or CT. Prevailing theories suggest that repetitive asymptomatic head trauma, concussion, and mTBIs result in damage to cortical and subcortical microstructures despite observable findings on conventional MRI being rare [40]. Several studies of white matter integrity using DTI support this assertion. Among long white matter tracts in the brain, the genu and body of the corpus callosum most consistently show evidence of microstructural changes associated with head trauma [26, 41]. Presumably, accumulated exposure to repetitive head trauma would therefore ultimately lead to brain tissue loss and measurable differences in brain volume compared to otherwise healthy individuals without repetitive head trauma. Multiple studies have compared groups across the adult lifespan with and without repetitive head trauma. Collision sport athletes are the most studied population.

### Professional Collision Sport Athletes

Former professional American football players and boxers represent the extreme of repetitive head trauma exposure and are a highly selected subgroup of collision sport athletes. As such, study samples are often small. Findings suggest that symptomatic (i.e., with cognitive and/or behavior and mood changes) former professional American football athletes may have lower amygdala [42], hippocampus [42–44], cingulate gyrus [42], fronto-insular [43], and anterior temporal [43, 45] brain volumes than age-matched healthy controls without head trauma. A study of active and recently retired professional rugby players similarly found lower bilateral hippocampal and left amygdala volumes than controls; differences were attributed partially to alcohol use [46]. Hippocampal volume differences in particular may result from steeper age-related atrophy in those with repetitive head trauma [47]. Subcortically, lower thalamic volumes have been associated with earlier age of initiating American football participation among retired professionals [48]. The Professional Fighters Brain Health Study investigated 476 active and former professional fighters (boxers and mixed martial artists; 92% active fighters and otherwise healthy) compared to 63 unexposed controls and found lower thalamus and corpus callosum volumes among fighters [49].

Conversely, some studies of former professional American football and hockey athletes without objective cognitive impairment showed no brain volume differences compared to controls [50]. Soccer participation has also raised concerns for brain health because of exposure to headers and high concussion risk [2], and one small study of former professional male soccer players noted areas of lower cortical thickness in inferior parietal, temporal, and occipital cortices [51]. Data from multi-site studies targeting former professional collision sport athletes, like DETECT (Diagnosing and Evaluating Traumatic Encephalopathy using Clinical Tests) and DIAGNOSE CTE (Diagnostics, Imaging, and Genetics Network for the Objective Study and Evaluation of Chronic Traumatic Encephalopathy), are expected to advance development of clinically applicable neuroimaging biomarkers.

### Non-Professional Collision Sport Athletes (High School, Collegiate)

Collegiate and high school collision sport athletes better represent general athlete population exposure levels to repetitive head trauma. Typically, these individuals have less overall lifetime head trauma exposure than professionals given the earlier “retirement” from their sport. The Concussion Assessment, Research, and Education (CARE) Consortium is a national multi-site study of sport-related head trauma (concussion and repetitive asymptomatic exposure) [52] that has produced several recent reports on structural brain changes in active collegiate athletes [53–55]. Brett and colleagues found that active, healthy collision sport athletes showed an association of more years of sport participation—a proxy for cumulative head trauma exposure—with lower thalamic volumes [56]. This effect was not observed in non-contact sport athletes. In a separate smaller study of active collegiate American football players, cortical thickness was lower than controls in several frontal lobe regions, but only in American football players who *also* had a history of symptomatic concussion [57]. This suggests a potential moderating or synergistic effect of symptomatic events with repetitive asymptomatic trauma on brain volume development or tissue loss. A longitudinal investigation of collegiate American football players found several regions of lower volume compared to non-contact athletes (volleyball) at baseline [58]. However, the American football athletes paradoxically exhibited *less* grey matter volume loss and cortical thinning over up to 4 years of follow-up than the non-contact athletes. This was interpreted as a potential pathologic disruption to normal neurodevelopmental and myelination dynamics seen in adolescence and early adulthood [59].

Most American football participants do not play past high school. Data indicate that older adults with prior high school level exposure are indistinguishable on brain health metrics from older adults without prior head trauma exposure [60–62]. Former high school football players reporting multiple symptomatic concussions did not have significantly different brain volumes than former high school players without prior concussion, but no pure control group without head trauma exposure was included [63]. While our focus in this review is on structural MRI, multiple studies of active high school and collegiate American football athletes have reported evidence for altered white matter microstructure and functional connectivity associated with repetitive head impacts even in the absence of symptomatic injuries [26, 41, 64–69]. The chronicity of these changes, relation to volume loss, and relevance for later-life brain health remain unclear.

### Military Service and Repetitive Blast Exposure

Few studies have directly evaluated brain volume changes associated with repetitive blast exposure in military servicemembers, which contrasts the frequent study of acute and chronic outcomes of discrete TBI events [70–72]. Breachers are a unique subpopulation of military servicemembers (and of law enforcement) frequently exposed to repeated, low-intensity blasts during training and active duty. One small study of 20 breachers reporting between 100 and 35,000 estimated career blast exposures found *greater* cortical thickness in occipital lobe and default mode network regions (medial frontal, medial temporal, inferior parietal, precuneus, posterior cingulate cortices) compared to unexposed controls [73]. Authors speculated that this finding may reflect alterations in cortical myelination, intracortical connections, or glial scarring at the gray-white matter junction that image processing pipelines miscalculate when distinguishing between tissue types [73]. Baseline (i.e., pre-exposure) group structural differences, as well as other factors that influence volumetric *measurements* also cannot be ruled out. Another small study of 10 military veterans reporting frequent low-level blast exposures found no volumetric differences from unexposed controls, but noted areas of nonspecific white matter hyperintensity signal in 5 of the 10 blast-exposed veterans [74].

### Cavum Septum Pellucidum: A “Canary in the Coal Mine” for Repetitive Head Trauma-Related Neurodegenerative Disease

**Fig. 1:**
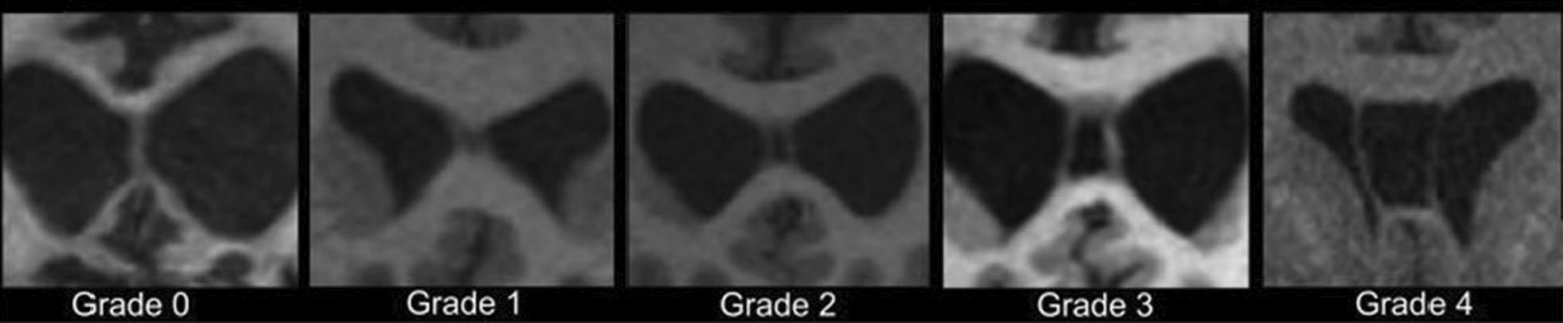
Enlarged views of representative T1 coronal images for each “grade” of cavum septum pellucidum (CSP). A “Grade 0” septum pellucidum appears crisp without any evidence of cyst or separation (CSP absent). “Grade 1” CSP shows slight interior hypointensity that is not clearly CSF signal intensity (septum unclear/CSP equivocal). Grades 2–4 show clear evidence of CSF signal between separated leaves of the septum pellucidum. The degree of separation between the leaves is used to assign a grade of 2–4: Grade 2 CSP is not wider than the septum, Grade 3 CSP is wider than the septum but less than half the intraventricular width, and Grade 4 CSP is greater than half the intraventricular width. Grading is based on the coronal slice that shows the greatest evidence of separation of the leaves of the septum pellucidum. *Figure and caption * *adapted from Gardner *et al*. 2016, J Neurotrauma, 33(1):157–61 (permissions pending review and acceptance of manuscript)*

Cavum septum pellucidum (CSP) refers to separation of the septum pellucidum usually best observed on coronal view of conventional T1 MRI as hypointense (CSF filled) space between the two septal leaflets (Fig. 1). CSP was first detectable using air encephalography in the 1930s. Forster (1933) recognized CSP in a patient who suffered a brain injury and later died, at which point CSP was confirmed at autopsy. A smattering of additional cases was reported in subsequent decades before Spillane’s case series of “Five Boxers” in 1962 [75], wherein he observed septum pellucidum damage in three of the five boxers. One year later, Mawdsley & Ferguson reported CSP in 7 of 10 ex-boxers with chronic neurologic impairment [76]. The authors contended that “Changes in septum pellucidum probably cause no neurological deficit. Radiologic demonstration of a cavum, by implicating boxing as its probable cause, is helpful diagnostically” [76]. Growing evidence over the following 60 years largely supports Mawdsley’s and Ferguson’s prescient conclusions.

CSP has been observed at a striking, but variable, rate within repetitive head trauma cohorts in recent years. Researchers have questioned the sensitivity and specificity of CSP to repetitive head trauma exposure, but the frequency with which this phenomenon occurs in such cases warrants attention. Two 2016 publications reported higher rates of CSP in clinically impaired, former professional American football players. The first study showed CSP was more frequent compared to cognitively impaired, matched case-controls without prior head trauma (16/17, 94% vs. 3/17, 18%) [77]. The second study found higher rates compared to healthy, former noncontact sport athletes (66/72, 92% vs. 8/14, 57%) [78]. In both studies, CSP length was also significantly longer in the symptomatic former American football groups. Within the large Professional Fighter Brain Health Study cohort, 53% of 499 active and former fighters had CSP compared to 17% of 62 unexposed controls. CSP length was again significantly greater in the former fighters [79]. The high frequency of CSP in repetitive head trauma cohorts, particularly those experiencing neurologic changes, has prompted proposals that CSP may be a neuroimaging biomarker for CTE in living patients [16]. Autopsy case series reporting up to 65% of CTE cases with CSP [80, 81] support such recommendations, but the predictive value of CSP for identifying CTE remains unclear. The current evidence linking CSP to repetitive head trauma outweighs the evidence linking CSP to underlying CTE pathology as currently defined [34]. A key gap in the repetitive head trauma-CSP literature is a large-scale evaluation of CSP in asymptomatic, clinically normal individuals with repetitive head trauma. This would inform relevance of CSP to clinical symptoms or potential prognostic risk for later development of neurodegenerative disease.

CSP has also been observed in other patient populations and in variable rates among otherwise healthy individuals. A meta-analysis of CSP prevalence in psychiatric populations found 1.4 × greater likelihood of CSP compared to psychiatrically healthy controls and almost 2 × greater likelihood of a large CSP (≥ 6 mm length), though there was significant heterogeneity across included studies [82]. Schizophrenia, in particular, is classically linked to higher rates of CSP but data suggest that this association may be limited to risk for larger CSP than presence per se [83]. Similar associations with CSP enlargement have been noted in adolescent-onset opiate dependence [84] and obsessive–compulsive disorder [85], though data are mixed [86]. It remains unclear whether CSP presence is congenital, develops over time, or is directly and meaningfully associated with clinical symptoms. Limited evidence from a small subset of serially imaged boxers suggests CSP develops and increases in size in the course of repetitive trauma exposure [87, 88]. It is also essential to consider that other populations with high rates of CSP, like those with psychiatric illness, are at significantly higher risk of lifetime head trauma exposure [89–91]. As discussed previously, studies not focused on head trauma may not assess participants for possible exposure or may rely on insensitive methods to rule out exposure. Anecdotally, CSP with fenestrations or a “ratty” appearance may imply a traumatic etiology as some have speculated rapid acceleration-deceleration or fluid percussion force causes shearing of the two septal leaflets.

### Structural White Matter Abnormalities

White matter susceptibility to traumatic forces underscores the potential for findings on clinically relevant structural MRI sequences. Active collegiate hockey players were found to have a similar number of white matter hyperintensity (WMH) counts to controls on T2/fluid attenuated inversion recovery (FLAIR) imaging [92]. However, WMH lesions in the athlete group were located more closely to the grey-white matter junction and to sulcal depths, regions associated with early CTE pathology in affected brains. There was also a slight predominance for the frontal lobes (75% of all lesions) relative to controls (56%). Lesion counts did not change over the course of a single hockey season and did not increase acutely after concussion. In a small study of collegiate American football players, a subset of athletes exhibited general decreased susceptibility weighted imaging (SWI) signal after one season of participation, which was interpreted as potential evidence for asymptomatic, trauma-related microbleeds [64]. Alosco et al. reported higher frequency of white matter signal abnormalities on high-resolution T1 in former professional American football athletes than controls, the number of which correlated with the estimated amount of head trauma exposure throughout their playing career [93]. To date, there is less research using clinically available MRI sequences to characterize white matter abnormalities in repetitive head trauma cohorts than exists for acute brain injury patients.

It is unclear whether these structural white matter findings reflect neurodegenerative changes, especially in younger, asymptomatic participants. However, several studies document cerebrovascular pathophysiology associated with repetitive head trauma, including altered cerebral perfusion dynamics [64, 94–97] and blood–brain-barrier dysfunction [98, 99], which may produce white matter signal abnormalities on MRI. In CTE cases, more severe white matter rarefaction is associated with greater exposure to repetitive head trauma, severity of neurofibrillary tangle deposition, and likelihood of developing dementia [32].

### Take-Home Points on Structural MRI in Repetitive Head Trauma

No characteristic atrophy pattern has emerged as specific to repetitive head trauma exposure. There are somewhat consistent findings of lower medial temporal and subcortical (thalamus, corpus callosum) volumes in repetitive head trauma cohorts compared to controls across the lifespan, though interpreting group level volume differences in younger, active athletes is complicated by neurodevelopmental dynamics. We cannot readily attribute volumetric differences to a specific neurodegenerative pathology (e.g., CTE) but susceptibility of these regions to traumatic forces implicates repetitive head trauma regardless of the underlying pathophysiologic process. Collective evidence indicates that presence of CSP in clinically impaired adults with repetitive head trauma exposure should increase suspicion that the presence of any neurodegenerative disease, whether CTE or otherwise, is attributable at least in part to the patient’s repetitive head trauma. Emerging research suggests repetitive head trauma exposure may also lead to white matter alterations observable on clinically available structural MRI sequences (i.e., T2/FLAIR). White matter pathologies seen in CTE and their direct relevance for clinical impairment [80, 100–102] strongly implicate the importance of further studying neuroimaging modalities that characterize white matter changes in repetitive head trauma cohorts.

## Positron Emission Tomography (PET) Metabolic and Molecular Neuroimaging

### FDG-PET Neuroimaging of Repetitive Head Trauma

FDG-PET (2-deoxy-2-(18F)fluoro-deoxyglucose) provides in vivo evidence of the severity and spatial distribution of changes in brain metabolism presumed to represent altered synaptic activity. Few studies have used FDG-PET to evaluate participants with a history of repetitive head trauma. Two small studies of active and former boxers found lower FDG uptake (i.e., hypometabolism) in multiple but inconsistent regions including posterior cingulate [103], bilateral frontal lobes [104], parieto-occipital cortex [103], and the cerebellum [103]. Former American football players had significantly lower frontotemporal metabolism than controls [43]. In one study of military veterans, higher number of prior blast exposures correlated with lower cerebellar metabolism [105]. An antemortem PET-to-autopsy case report (CTE stage IV with hippocampal sclerosis) showed mild FDG hypometabolism corresponding with medial temporal and frontal atrophy. Medial temporal structures contained multiple degenerative protein aggregates, while frontal lobe pathology was predominantly CTE (see [106] and description of Patient #1 below).

### Aβ-PET Neuroimaging of Repetitive Head Trauma

In 2012, Florbetapir became the first Aβ-PET tracer approved by the U.S. Food and Drug Administration (FDA) for detecting moderate to frequent neuritic Aβ plaques, a core neuropathological feature of AD. Two similar radiotracers received approval shortly thereafter (Flutemetamol, Florbetaben). Widespread clinical implementation of Aβ-PET imaging remains limited in the U.S. and other countries due to lack of insurance reimbursement. However, emerging data from the Imaging Dementia – Evidence for Amyloid Scanning (IDEAS) study strongly support the relevance of Aβ-PET imaging in clinical management of cognitively impaired older adults [107]. Clinical feasibility of more routine Aβ-PET scans therefore may increase significantly, especially if tied to effective Aβ lowering therapeutic agents.

Most studies of repetitive head trauma do not specifically analyze associations with cortical Aβ burden. Instead, Aβ-PET often is used to rule out or identify comorbid AD. A negative Aβ-PET scan previously was proposed as a “positive biomarker” supporting a “Probable CTE” diagnosis [16] because Aβ plaques are not a diagnostic feature of CTE and their absence strongly suggests AD is not driving symptoms [34]. Among 11 living patients with “Probable CTE,” 2 were Aβ-PET positive and also showed the most severe atrophy plus tau PET signal [43]. In brains with CTE at autopsy, Aβ deposition is a common co-pathology (> 90% of former professional American football players with advanced CTE) [5], occurs at an accelerated rate, and preferentially affects the depths of cortical sulci [108]. Aβ plaques in CTE usually are diffuse rather than neuritic, which may explain lower affinity of Aβ-PET tracers.

Acute brain injury has been linked to upregulation of amyloid precursor protein, which is cleaved to form Aβ polypeptides [109–112]. Studies evaluating Aβ-PET acutely after TBI inconsistently note presence of cortical Aβ plaques [25]. One autoradiography study reported white matter accumulation of Aβ and amyloid precursor protein, but no binding of Aβ-radiotracer (Pittsburgh Compound B; PIB), which aligned with their finding of no differences in white matter PIB binding between TBI patients and controls [113]. Across two studies of moderate-severe TBI patients compared to controls, one showed greater cortical grey matter and striatum binding (< 1 yr post-TBI) [113] and one showed greater posterior cingulate and cerebellum binding (> 1 yr post-TBI) [114]. Conversely, a recent investigation of remote head trauma exposure (both mild TBI and a subset with repetitive asymptomatic exposure) found no association with later-life cortical Aβ burden using PET in clinically normal older adults [115]. Others similarly have reported a lack of association between remote, mild head trauma exposure and cortical Aβ burden [116–118].

### Tau-PET Neuroimaging of Repetitive Head Trauma

#### FDDNP

Most early tau-PET studies in repetitive head trauma patients used the FDDNP radiotracer. FDDNP binding properties severely limit its sensitivity and specificity to CTE pathology. FDDNP binds to different protein aggregates that form beta-pleated sheets (Aβ plaques, tau tangles, prion proteins, and others), has poor reproducibility, and has a low signal-to-noise ratio [119–122]. Regardless, several early studies found group-level differences between repetitive head trauma participants and controls in the spatial pattern and degree of FDDNP tracer uptake [123, 124]. Data indicated that groups of blast-exposed veterans and former professional American football players, albeit often with small numbers, showed FDDNP binding in white matter and subcortical structures [123] along with limbic and brain stem regions [123, 125]. Binding patterns seemingly differed from AD cases as well. One case study reported a former American football player diagnosed with CTE at autopsy (also with frequent neuritic Aβ plaques) who underwent FDDNP-PET imaging about 4 years before death. The report showed that FDDNP binding levels correlated with the amount of tau deposition in the brain at autopsy [126]. However, the FDDNP radiotracer is not FDA approved and there is no support for clinical utility.

##### Flortaucipir (FTP)

FTP was developed to detect paired helical filament tau in neurofibrillary tangles characteristic of AD (now FDA approved). Multiple investigations consistently support FTP use for differentiation of AD from controls and non-AD tauopathies [127–129], but there is limited comparison to CTE. There were high hopes that the science of CTE and repetitive head trauma biomarker development could ride the wave of extremely promising research demonstrating strong affinity of the FTP tracer to AD tau [127]. Excitement over potential CTE diagnosis stemmed from known similarities in phosphorylated tau isoforms between AD and CTE – mixed 3-repeat/4-repeat tau tangles with paired helical filament structures. However, an autoradiography study showed that FTP only weakly bound to brain tissue with dense CTE pathology compared to its strong binding to tissue with AD tau [130], again suggesting limited potential for sensitive or specific CTE detection. Newly identified differences in fibril folding microstructure between CTE and AD tau may explain differences in tau radiotracer binding affinity [131].

FTP exhibits “off-target” (i.e., non-tau related) binding to choroid plexus (often complicating medial temporal signal interpretation) [132, 133], caudate, putamen, pallidum, thalamus, and white matter [133–135], and cortically in some cases of tau-negative neurodegenerative disease [127]. An early case report speculating that FTP binding in the basal ganglia reflected a “novel variant” of CTE likely represented off-target tracer binding [133, 134, 136]. More recently, Stern et al. reported a group-level comparison of 26 predominantly Aβ-PET negative former professional American football athletes to 31 controls and found higher FTP binding in medial temporal, parietal, and superior frontal lobes [137]. Degree of FTP uptake correlated with number of years participating in American football, but there was no association with cognitive outcomes [138]. Lesman-Segev et al. compared 11 clinically impaired TES patients to clinically impaired, biomarker-confirmed AD patients and unexposed, Aβ-PET negative, clinically normal controls [43]. There was mildly elevated FTP binding in frontotemporal regions of TES patients relative to unexposed controls, and no regions with higher FTP signal than the AD group. Some patients exhibited FTP binding in a non-contiguous “dot-like” pattern, similar to data reported in a small group of veterans with history of multiple low-level blast exposures [74]. This pattern is also observed in some healthy controls [43] and may simply represent noise or imaging artifact [43, 127].

A recent case report compared antemortem FTP binding in a former professional American football player to neuropathology observed at autopsy [106]. The patient had severe CTE (stage IV) and hippocampal sclerosis without comorbid AD. FTP uptake overlapped well with CTE tau pathology in the inferior temporal lobe and juxtacortical frontal white matter, but there was weak FTP uptake on PET imaging in several areas of the brain with dense CTE tau deposition at autopsy and confirmed off-target binding subcortically.

The recent release of the FDA’s label for FTP provides essential context for application in clinical settings. FTP scans are indicated for estimating the density and distribution of aggregated tau neurofibrillary tangles in adults with cognitive impairment who are being evaluated for AD. “Positive” scans show visually apparent increased neocortical tracer uptake in the posterolateral temporal, occipital, or parietal/precuneus regions, with or without frontal uptake. *FTP is not indicated for use in the evaluation of patients for CTE*. This does not inherently rule out the potential clinical utility of FTP-PET scans for patients with a history of repetitive head trauma if their clinical profile raises suspicion for AD. In this scenario, a “positive” FTP scan would implicate underlying Alzheimer’s disease as contributing to cognitive impairment (especially if accompanied by elevated Aβ PET), but would not rule out comorbid CTE. A “negative” FTP scan might *increase* the likelihood that CTE is driving cognitive symptoms, contrary to prior research criteria proposing that positive tau PET findings fulfill the biomarker-based requirement for “Probable CTE” [16].

##### Other Tau PET Tracers and Considerations for Future Development

Several additional tau PET tracers exist, and others are rapidly being developed [139], but most thus far have rarely been used in repetitive head trauma research. PBB3 is a family of tau PET compounds that appears to bind tau aggregates consisting of all isoforms [140, 141]. Takahata and colleagues found that patients with TES showed higher [11C]-PBB3 binding in white matter than individuals with single-event TBI [142]. Binding to tau lesions at the depths of neocortical sulci (suggesting CTE pathology) was confirmed via in vitro assays [142]. The second-generation MK-6240 tracer is a highly selective paired helical filament tau tracer with less off-target binding in the brain, but with off-target meningeal binding [143, 144]. A recent case report of a former Australian rules football athlete described in vivo MK-6240 cortical uptake in a pattern resembling the spatial distribution of CTE (bilateral superior frontal and medial temporal regions) and distinct from a typical AD pattern [145]. However, limited autoradiographic evidence suggests MK-6240, like FTP, may have high affinity for AD tau tangles but not CTE tau [143]. Additional validation work is necessary. Other tau tracers include RO-948, PI-2620, and GTP-1. These tracers are derivatives of FTP and thus likely have similar binding characteristics, but this has not been tested empirically.

Temporal dynamics of the underlying disease process may also be particularly relevant for developing a diagnostic PET biomarker for CTE. For example, FTP binds neurofibrillary tangles and autopsy studies suggest that a “positive” scan requires advanced AD tau pathology (Braak stage V-VI). Early-stage CTE involves sparse neurofibrillary tangle deposition often located at brain/CSF interfaces where PET signal can be washed out by partial volume effects. It is therefore likely that a CTE-specific PET tracer will be sensitive only to relatively advanced pathology (i.e., CTE Stage III-IV). Further complicating matters in CTE, which is a mixed 3R/4R tauopathy, the 4R tau isoform may be much more prevalent than the 3R isoform earlier in the disease process before shifting towards deposition of 3R tau and fully formed neurofibrillary tangles [146]. Astrocytic tau inclusions are also a prominent feature of CTE despite being insufficient for formal diagnosis [34]. Recent work showed that the tau tangles within neurons are a mix of 3R and 4R isoforms while astrocytes predominantly contain 4R tau [146, 147].

### Take-Home Points on FDG-PET, Aβ-PET, Tau-PET Neuroimaging

FDG-PET study findings implicate inconsistent brain regions, which is not surprising given the heterogeneous underlying diseases within clinically impaired repetitive head trauma cohorts. The main utility of Aβ-PET in repetitive head trauma research currently rests on ruling out concomitant AD pathology. Collective findings thus far unfortunately suggest limited utility of well-studied AD tau PET radiotracers for identifying CTE. A radiotracer sensitive and specific to CTE-tau must be developed and likely must account both for variations in relative presence of 3R versus 4R tau isoforms at different disease stages (e.g., mild or severe) and in different cell types (neurons versus astrocytes). Appreciation for off-target (i.e., non-tau) binding properties and nonspecific binding patterns (non-contiguous, “dot-like”) is critical to avoid potential false-positive diagnoses.

## Clinico-Pathologic Examples of Clinically Suspected CTE Patients With and Without CTE Pathology at Autopsy

**Fig. 2:**
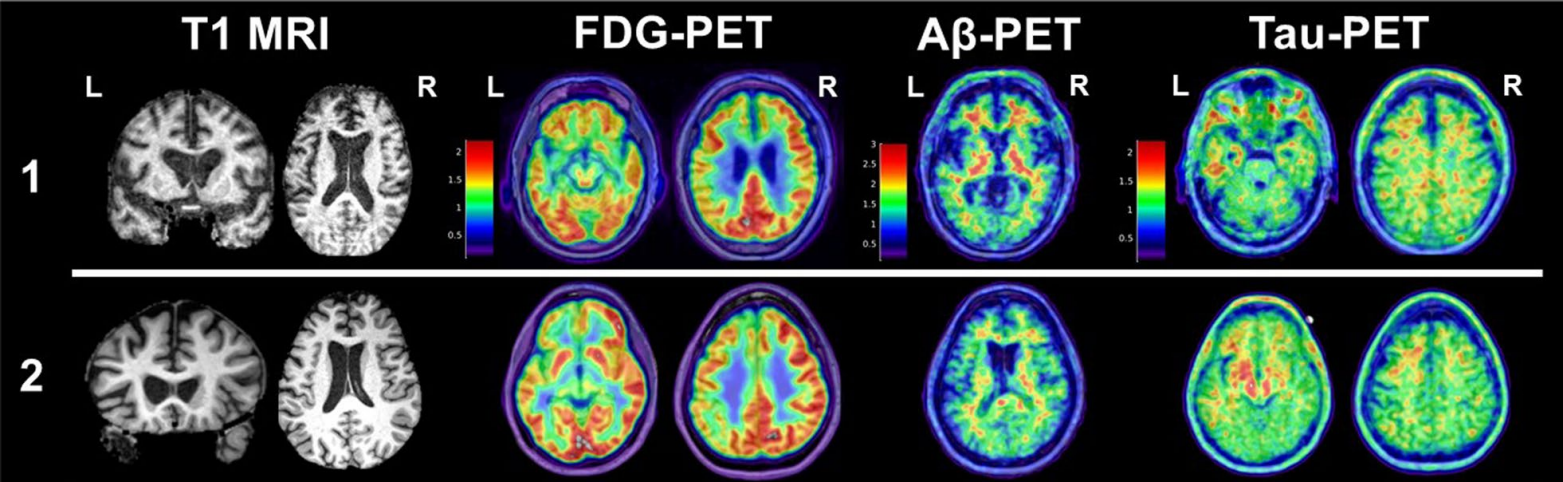
Representative slices of antemortem structural T1 magnetic resonance imaging (T1 MRI), FDG-PET, Aβ-PET (PIB), and tau-PET (FTP) for 2 clinically impaired adults with prior repetitive head trauma meeting criteria for “Probable CTE” (see text for case descriptions). Patient #1 had a primary neuropathologic diagnosis of CTE (Stage IV) with contributing hippocampal sclerosis and left subiculum microinfarct (no AD pathology observed). Patient #2 had a primary neuropathologic diagnosis of FTLD-tau (corticobasal degeneration) with contributing hippocampal sclerosis and unclassifiable FTLD-TDP-43 inclusions (no CTE or AD pathology observed). For FDG-PET, cooler colors represent areas of decreased glucose uptake (hypometabolism). For PIB-PET, warmer colors represent increased tracer uptake. A positive Aβ-PET scan is represented by increased tracer uptake diffusely throughout the cortex. In both patients, Aβ tracer uptake is restricted to the white matter, which is considered non-specific and represents a negative Aβ-PET scan. For FTP-PET, warmer colors represent areas of increased tracer binding. A “positive” scan for AD neurofibrillary tangles requires contiguous neocortical uptake in the posterolateral temporal, occipital, or parietal/precuneus regions with or without frontal uptake. Neither patient showed a typical AD pattern of FTP tracer uptake, while both showed evidence of nonspecific, scattered frontotemporal uptake and non-specific increased signal in the basal ganglia. Slices were chosen to highlight cavum septum pellucidum (T1 MRI) or representative areas of hypometabolism (FDG-PET) and Aβ/tau tracer uptake. Additional brain slices for PET scans from each case are provided in supplemental material

Here we present two research participants from the UCSF Memory and Aging Center’s Alzheimer’s Disease Research Center. Both patients were evaluated and discussed by multidisciplinary consensus conference after comprehensive neurological and neuropsychological evaluations. Each patient also underwent antemortem, multimodal neuroimaging obtained prior to death and autopsy. Both patients met 2014 TES criteria for “Probable CTE.” We also retroactively applied the recent 2021 TES diagnostic framework, which resulted in one patient clinically diagnosed with “Probable CTE” and one patient diagnosed with “Possible CTE.” Neuroimaging modalities included structural MRI, FDG-PET, Aβ-PET (PIB), and tau-PET (FTP). We provide summaries of neuroimaging and neuropathologic findings to demonstrate potential limitations of existing neuroimaging techniques for identifying CTE pathology. Figure 2 and the Additional file 1: supplementary figure show slices from antemortem neuroimaging.

### Patient #1

The first patient (described in detail elsewhere [106]) was a 72-year-old former professional American football player with a 17-year participation history that began at age 14. His primary playing position was linebacker. He reported a 13-year symptom progression with initial changes in behavior and mood (irritability, anger outbursts, social withdrawal, depression), memory, and executive function. He later developed parkinsonism and seizures. Consensus clinical diagnosis was TES-mixed variant. Applying the 2021 TES diagnostic framework, Patient #1 qualifies for “Probable CTE” based on presence of ≥ 11 years of American football exposure, delayed symptom onset, emergence of motor signs, ≥ 1 psychiatric feature, and neurobehavioral dysregulation.

Antemortem structural MRI (age 68) showed cavum septum pellucidum (Grade 2; 12 mm length). There was predominantly anterior and ventral atrophy including bilateral limbic and medial temporal regions and the left middle frontal lobe. PET scans were obtained at the same visit as structural MRI. FDG-PET showed mild hypometabolism in medial temporal and frontal lobes. Aβ-PET was negative. Tau-PET showed tracer uptake in a non-AD pattern including nonspecific scattered frontotemporal binding. The patient fulfilled 3 (only 1 required) of the biomarker-based criteria from the 2014 TES criteria for Probable CTE (CSP, negative Aβ-PET, cortical atrophy) and also had some evidence suggesting abnormal tau-PET.

The primary neuropathologic diagnosis was CTE (Stage IV). Immunohistochemical analysis showed frequent clusters of tau-immunoreactive neurons and astrocytes in perivascular spaces at depths of sulci located in cortical, subcortical, and spinal cord regions. The most affected cortical regions were frontal, temporal, and limbic structures. There was also focal hippocampal sclerosis (left; CA1) and a left subiculum microinfarct. Additional findings included limbic argyrophilic grain disease, limbic-predominant age-related TDP-43 encephalopathy (LATE; stage 2), aging-related tau astrogliopathy, and mild arteriolosclerosis. There was no evidence of Aβ pathology.

### Patient #2

The second patient was a 49-year-old former collegiate American football player with an 8-year participation history that began at age 12. His primary playing position was quarterback. He was also a professional motorcyclist and racecar driver wherein he suffered at least 3 additional mild TBIs after his American football career. He reported an 8-year symptom progression initially presenting with apathy, loss of empathy, social disinhibition, and compulsive behaviors. He later developed memory loss and language difficulties. Consensus clinical diagnosis was behavioral variant frontotemporal dementia. He also fulfilled criteria for TES-behavioral/mood variant. Applying the 2021 TES diagnostic framework, Patient #2 qualifies for “Possible CTE” based on ≥ 5 but < 11 years of American football exposure, delayed symptom onset, ≥ 1 psychiatric features, and neurobehavioral dysregulation.

Antemortem structural MRI (age 47) showed cavum septum pellucidum (Grade 1; 5 mm length). There was prominent left frontoparietal and subcortical atrophy including bilateral thalamus and left caudate. PET scans were obtained at the same visit as structural MRI. FDG-PET showed severe medial frontal, thalamic, and basal ganglia hypometabolism also affecting parietal and temporal lobes. Aβ-PET was negative. Tau-PET showed tracer uptake in a non-AD pattern including moderate frontotemporal white matter and basal ganglia binding with left predominance. The patient fulfilled 3 of the biomarker-based criteria from the 2014 TES criteria for Probable CTE (CSP, negative Aβ-PET, cortical atrophy) and also had evidence suggesting abnormal tau-PET.

The primary neuropathologic diagnosis was FTLD-tau (corticobasal degeneration). Immunohistochemical analysis showed frequent neuronal cytoplasmic inclusions, tau-positive ballooned neurons, coiled bodies, and variably dense astrocytic plaques, as well as severe subcortical white matter tauopathy. There was also widespread TDP-43 pathology with diverse inclusion types usually, but not always, in a pattern resembling the tauopathy (particularly prominent in medial temporal limbic structures and accompanied by severe hippocampal sclerosis affecting all sectors of Ammon’s horn). There was no evidence of CTE or AD pathology.

### Clinically Suspected CTE Patient Summary

Both patients exhibited clinical decline several years after repetitive head trauma exposure and had multiple biomarkers suggesting underlying CTE pathology per prior research criteria [16]. Atrophic areas on structural MRI and regional hypometabolism on FDG-PET were largely consistent with the presenting clinical syndromes. Of note, tau PET in each participant showed abnormal, low-level tracer uptake in a clearly non-AD pattern. In the absence of tau PET, which is not widely available clinically, presence of CSP and negative Aβ-PET alone would likely have made both patients strong candidates for harboring significant CTE pathology. It remains unknown if or how Patient #2’s head trauma exposure contributed to the onset, progression, or symptom manifestation of non-CTE neuropathologic processes. Beyond the limitations of available neuroimaging modalities for reliably identifying CTE, these cases highlight the diversity of prospectively documented symptom presentations and underlying neurodegenerative diseases among clinically impaired patients with repetitive head trauma exposure.

## Future Considerations for Neuroimaging Research in Repetitive Head Trauma

### Structural Neuroimaging

Structural MRI remains an important and relatively accessible component of clinical evaluations for patients with suspected neurodegenerative disease, but usually images are reviewed qualitatively. Incorporating quantitative MRI methods and/or advanced sequences like DTI into clinical practice may improve sensitivity to head trauma-related brain changes. Systematically collecting different types of lifetime head trauma exposure, from repetitive asymptomatic impacts to severe TBI, will facilitate our understanding of brain changes on structural MRI attributable to head trauma. While elite level collision sport athletes represent an important study group, extending these efforts to the broader aging population will enhance generalizability and improve risk estimation along a wider spectrum of head trauma exposure.

### Other PET Radiotracers

While there is an understandable focus on in vivo CTE tau identification, measuring the degree and spatial distribution of neuroinflammation [100, 148–150] and synaptic dysfunction are other potentially interesting PET applications for repetitive head trauma [151–153]. The translocator protein (TSPO) is a mitochondrial membrane protein that is upregulated in activated microglia, astroglia, and macrophages. Several TSPO-PET ligands have been developed as in vivo markers of neuroinflammation. One study of TSPO-PET using the [11C]DPA-713 tracer found higher signal in former American professional football players than controls in bilateral medial and superior temporal regions [15]. New molecular targets for imaging activated microglia and astrocytes are currently under development [150]. To our knowledge, PET imaging of synaptic loss (e.g., synaptic vesicle glycoprotein 2A; SV2A-PET) [154, 155] has not been performed in repetitive head trauma cohorts. It is unclear if these approaches will be clinically meaningful in isolation for specific disease identification (e.g., differentiating CTE from AD or other neurodegenerative diseases), but they may prove valuable for unlocking pathophysiologic mechanisms linking repetitive head trauma to increased neurodegenerative disease risk in general.

### Sex-Specific differences in Neuroimaging of Repetitive Head Trauma

Sex differences in neuroimaging findings among repetitive head trauma cohorts are unknown. Existing studies almost exclusively focus on male-predominant groups of collision sport athletes (e.g., American football) and military veterans. Reported sex differences in head impact biomechanics [156–158], brain injury outcomes [159–161], and risk for neurodegenerative disease [162, 163] underscore the critical importance of studying the role of sex in repetitive head trauma outcomes. Large-scale, longitudinal cohorts like the Professional Fighters Brain Health Study [164], CARE Consortium [52], Chronic Effects of Neurotrauma Consortium [165], and longitudinal follow-up of the Transforming Research and Clinical Knowledge in TBI cohort [166] (i.e., TRACK-TBI LONG) offer strong potential for studying sex-specific outcomes in the association of repetitive head trauma with brain volume changes or molecular/metabolic alterations on PET imaging. Former participants in presumably high-risk female collision sports like soccer/futbol, ice hockey, rugby, mixed martial arts, etc. may be especially important study cohorts. Additionally, intimate partner violence survivors, who are usually female, are an often overlooked but critically important group to study [167]. Comprehensive evaluation of both repetitive, asymptomatic exposure and symptomatic brain injuries will be essential to these efforts.

## Concluding Remarks

Repetitive head trauma may increase risk for multiple neurodegenerative outcomes, with much recent focus on CTE. Structural MRI studies in repetitive head trauma cohorts do not clearly suggest a specific pattern of volume loss, though subcortical structures like the thalamus and corpus callosum and medial temporal limbic region appear susceptible to repetitive traumatic forces. Cavum septum pellucidum is much more common in clinically impaired repetitive head trauma populations than both clinically normal and impaired cohorts without head trauma exposure. Presence of cavum septum in clinically impaired adults with repetitive head trauma should increase suspicion that head trauma exposure contributed to the underlying disease. White matter abnormalities occasionally are observable on conventional clinical MRI and may be spatially distinct in repetitive head trauma populations, but more work characterizing these abnormalities is needed. FDG-PET studies have not identified a characteristic repetitive head trauma pattern, which likely reflects the diversity of underlying neuropathologies and associated clinical syndromes. Tau-PET remains a promising research avenue but will require development of CTE-tau specific radiotracers given the lack of support for current tracers with strong affinity for AD tau. PET imaging of Aβ plaques and AD tau tangles may still be clinically useful in ruling AD in or out. CTE is highly associated with prior repetitive head trauma. However, we caution against tenuous assumptions that CTE is present, or the sole or primary source of symptoms, in clinically impaired repetitive head trauma cohorts without other compelling clinical or biomarker data. Improving diagnostic precision for neurodegenerative disease within repetitive head trauma cohorts requires antemortem imaging-to-autopsy studies and development of other in vivo biomarkers sensitive to the effects of repetitive trauma on brain health.

## Supplementary Information

**Additional file 1**.

## Data Availability

Data sharing is not applicable to this article as no datasets were generated or analysed during the current study.

## Abbreviations

AD: Alzheimer’s disease
Aβ: Beta-amyloid
CARE: Concussion Assessment, Research, and Education Consortium
CSF: Cerebrospinal fluid
CSP: Cavum septum pellucidum
CT: Computed tomography
CTE: Chronic traumatic encephalopathy
DAI: Diffuse axonal injury
DETECT: Diagnosing and Evaluating Traumatic Encephalopathy using Clinical Tests
DIAGNOSE-CTE: Diagnostics, Imaging, and Genetics Network for the Objective Study and Evaluation of Chronic Traumatic Encephalopathy
DTI: Diffusion tensor imaging
FDA: Food & Drug Administration
FDDNP: 2-(1-{6-[(2-Fluoroethyl(methyl)amino]-2-naphthyl}ethylidene)malononitrile
FDG: Fluorodeoxyglucose
FLAIR: Fluid attenuated inversion recovery
FTP: Flortaucipir
IDEAS: Imaging Dementia – Evidence for Amyloid Scanning
LOC: Loss of consciousness
MRI: Magnetic resonance imaging
mTBI: Mild traumatic brain injury
NFT: Neurofibrillary tangle
PET: Positron emission tomography
PIB: Pittsburgh Compound B
PTA: Posttraumatic amnesia
SV2A: Synaptic vesicle glycoprotein 2A
SWI: Susceptibility weighted imaging
TBI: Traumatic brain injury
TES: Traumatic encephalopathy syndrome
TRACK-TBI: Transforming Research and Clinical Knowledge in TBI
TSPO: Translocator protein
WMH: White matter hyperintensity
