# Supplementary material for: Identifying degenerative effects of repetitive head trauma with neuroimaging: a clinically-oriented review
Source: Acta Neuropathol Commun. 2021 May 22;9:96. doi: 10.1186/s40478-021-01197-4 (PMC8141132; doi:10.1186/s40478-021-01197-4)

Patient #1

(Described elsewhere in detail: Mantyh WG, Spina S, Lee A, Iaccarino L, Soleimani-Meigooni D, Tsoy E, Mellinger TJ, Grant H, Vandevrede L, La Joie R, Lesman-Segev O, Gaus S, Possin KL, Grinberg LT, Miller BL, Seeley WW, Rabinovici GD. Tau Positron Emission Tomographic Findings in a Former US Football Player With Pathologically Confirmed Chronic Traumatic Encephalopathy. JAMA Neurol. 2020 Apr 1;77(4):517-521. doi: 10.1001/jamaneurol.2019.4509. PMID: 31904765; PMCID: PMC6990867.)

FDG-PET

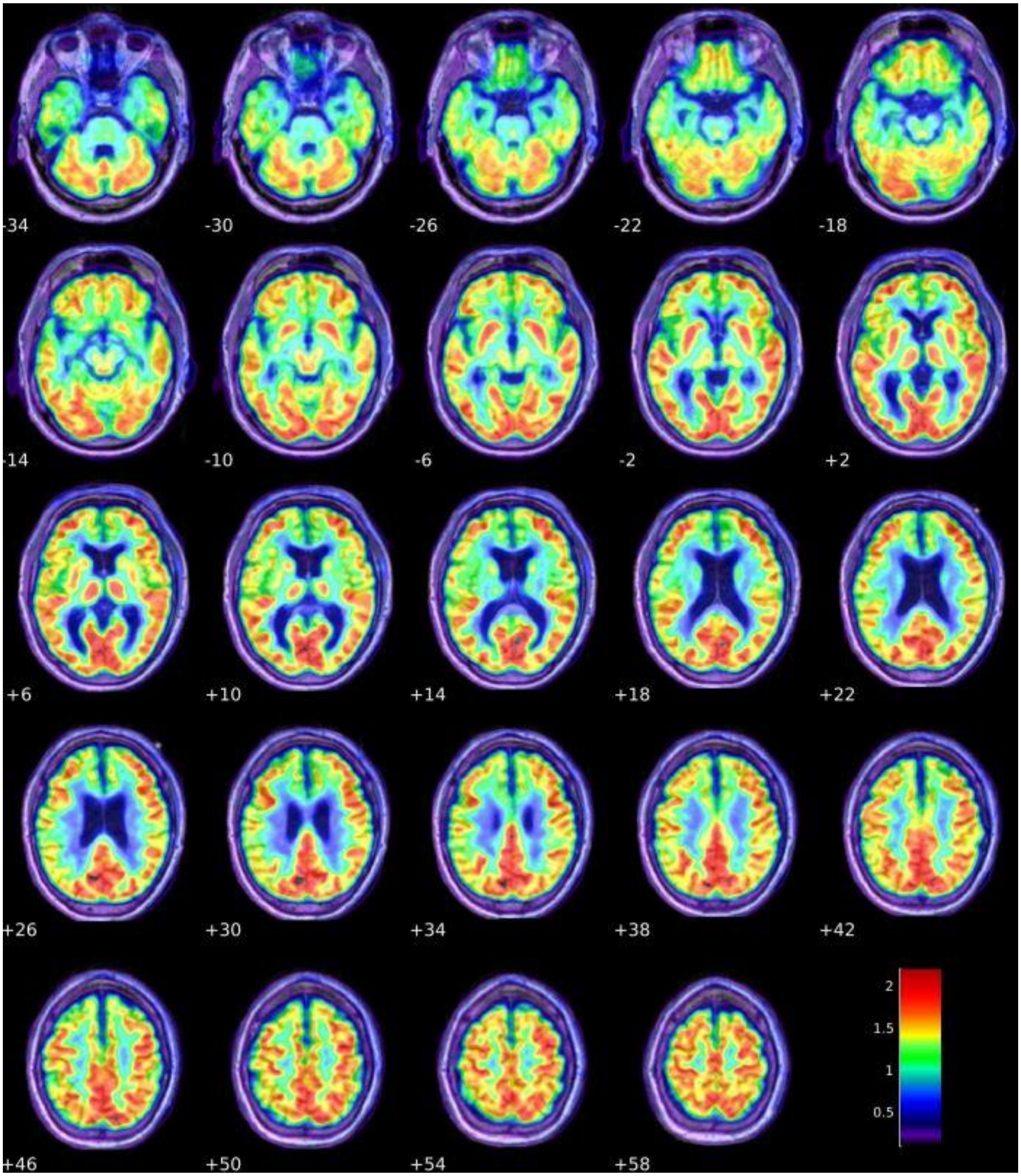

Patient #1

(Described elsewhere in detail: Mantyh WG, Spina S, Lee A, Iaccarino L, Soleimani-Meigooni D, Tsoy E, Mellinger TJ, Grant H, Vandevrede L, La Joie R, Lesman-Segev O, Gaus S, Possin KL, Grinberg LT, Miller BL, Seeley WW, Rabinovici GD. Tau Positron Emission Tomographic Findings in a Former US Football Player With Pathologically Confirmed Chronic Traumatic Encephalopathy. JAMA Neurol. 2020 Apr 1;77(4):517-521. doi: 10.1001/jamaneurol.2019.4509. PMID: 31904765; PMCID: PMC6990867.)

Aβ-PET (Pittsburgh compound B)

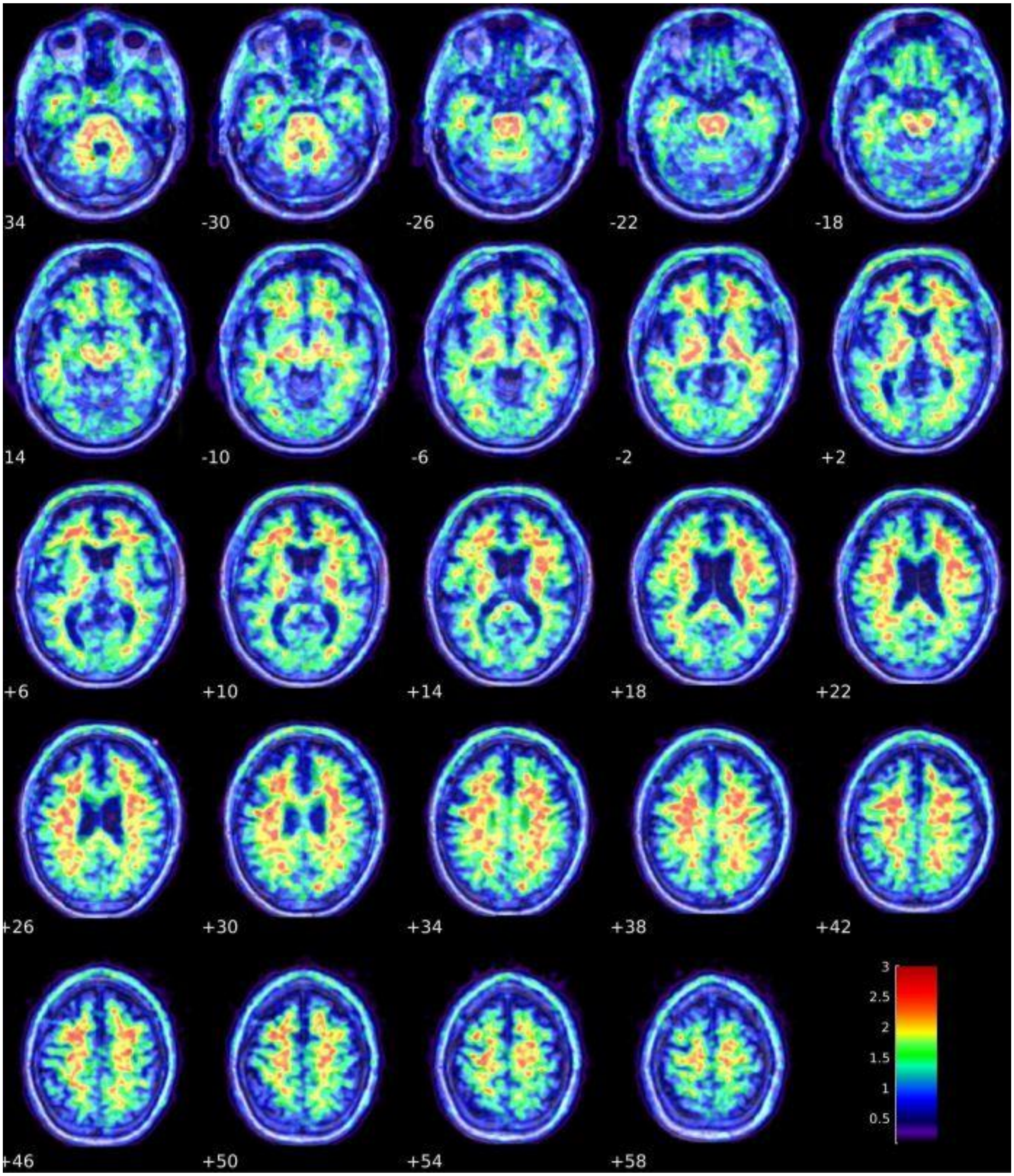

Patient #1

(Described elsewhere in detail: Mantyh WG, Spina S, Lee A, Iaccarino L, Soleimani-Meigooni D, Tsoy E, Mellinger TJ, Grant H, Vandevrede L, La Joie R, Lesman-Segev O, Gaus S, Possin KL, Grinberg LT, Miller BL, Seeley WW, Rabinovici GD. Tau Positron Emission Tomographic Findings in a Former US Football Player With Pathologically Confirmed Chronic Traumatic Encephalopathy. JAMA Neurol. 2020 Apr 1;77(4):517-521. doi: 10.1001/jamaneurol.2019.4509. PMID: 31904765; PMCID: PMC6990867.)

Tau-PET (Flortaucipir)

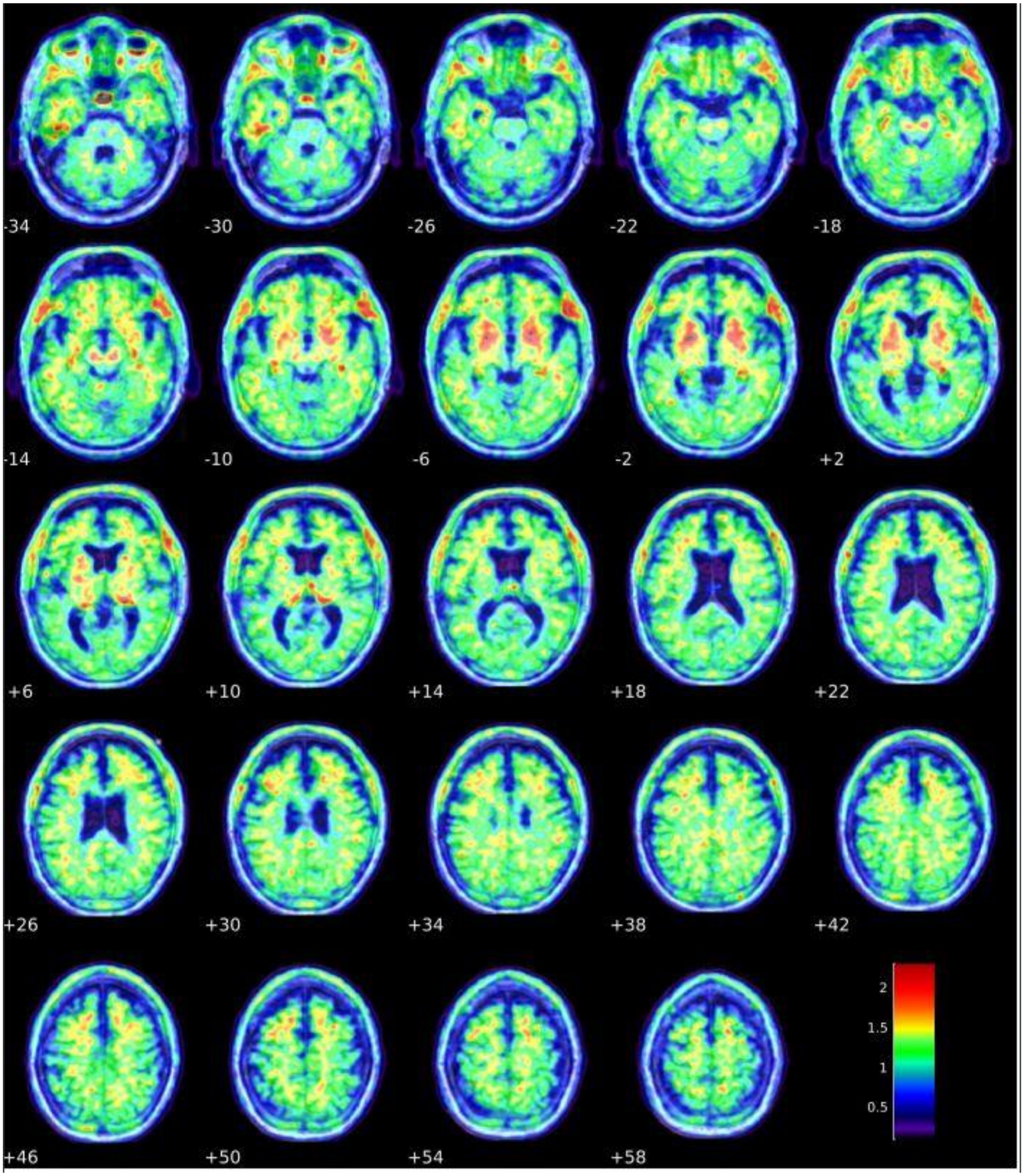

Patient #2

FDG-PET

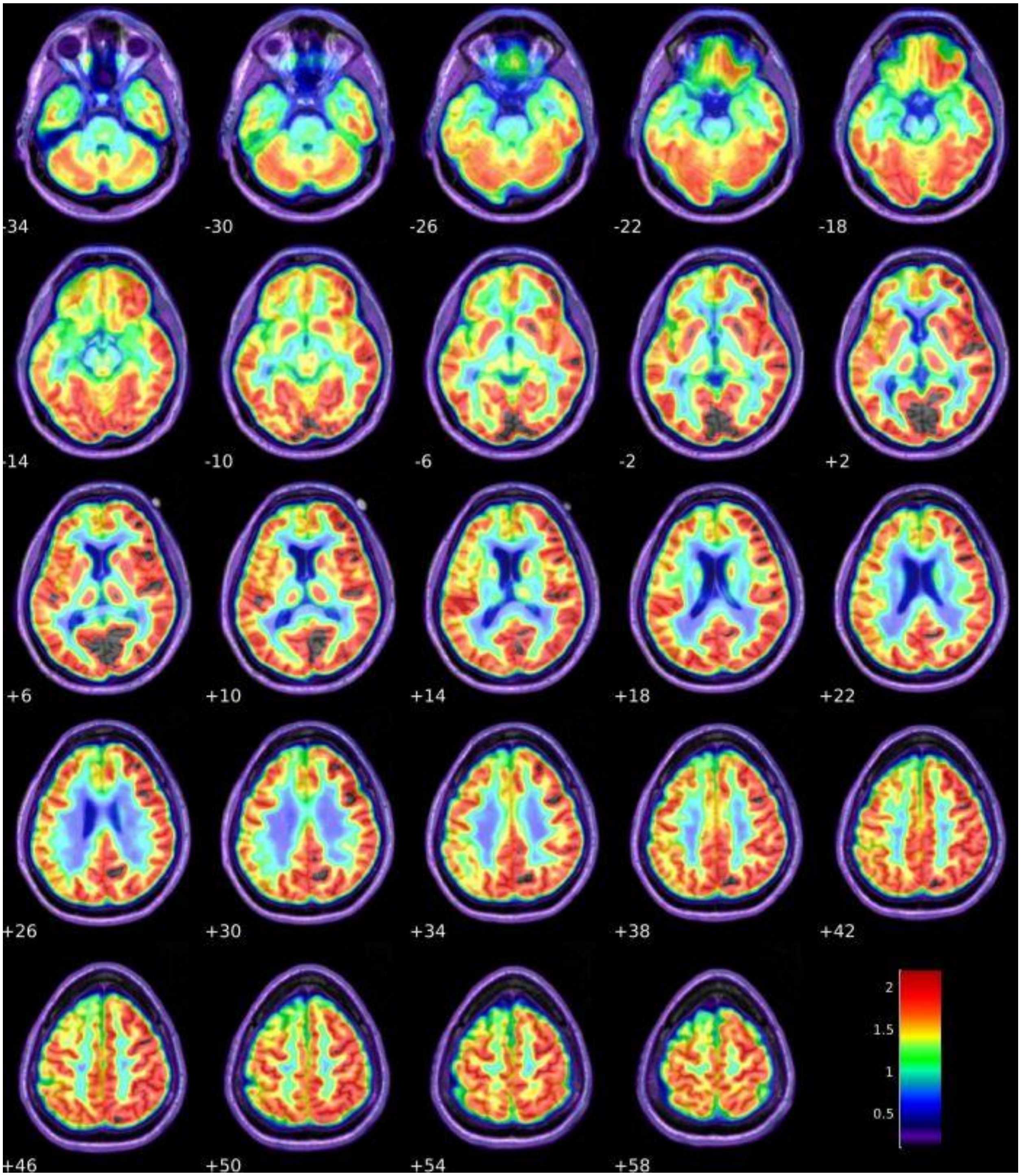

Patient #2

Aβ-PET (Pittsburgh compound B)

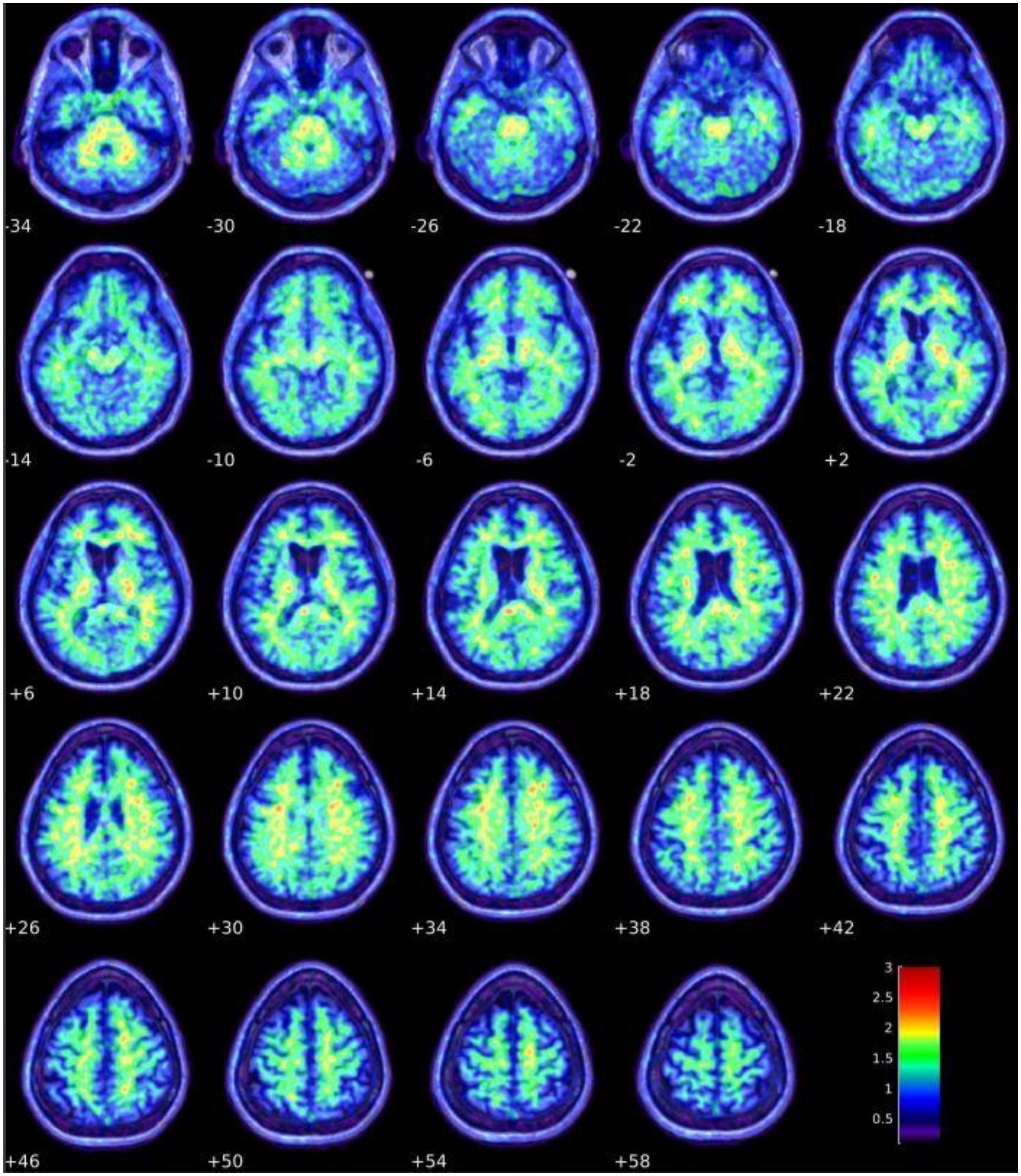

Patient #2

Tau-PET (Flortaucipir)

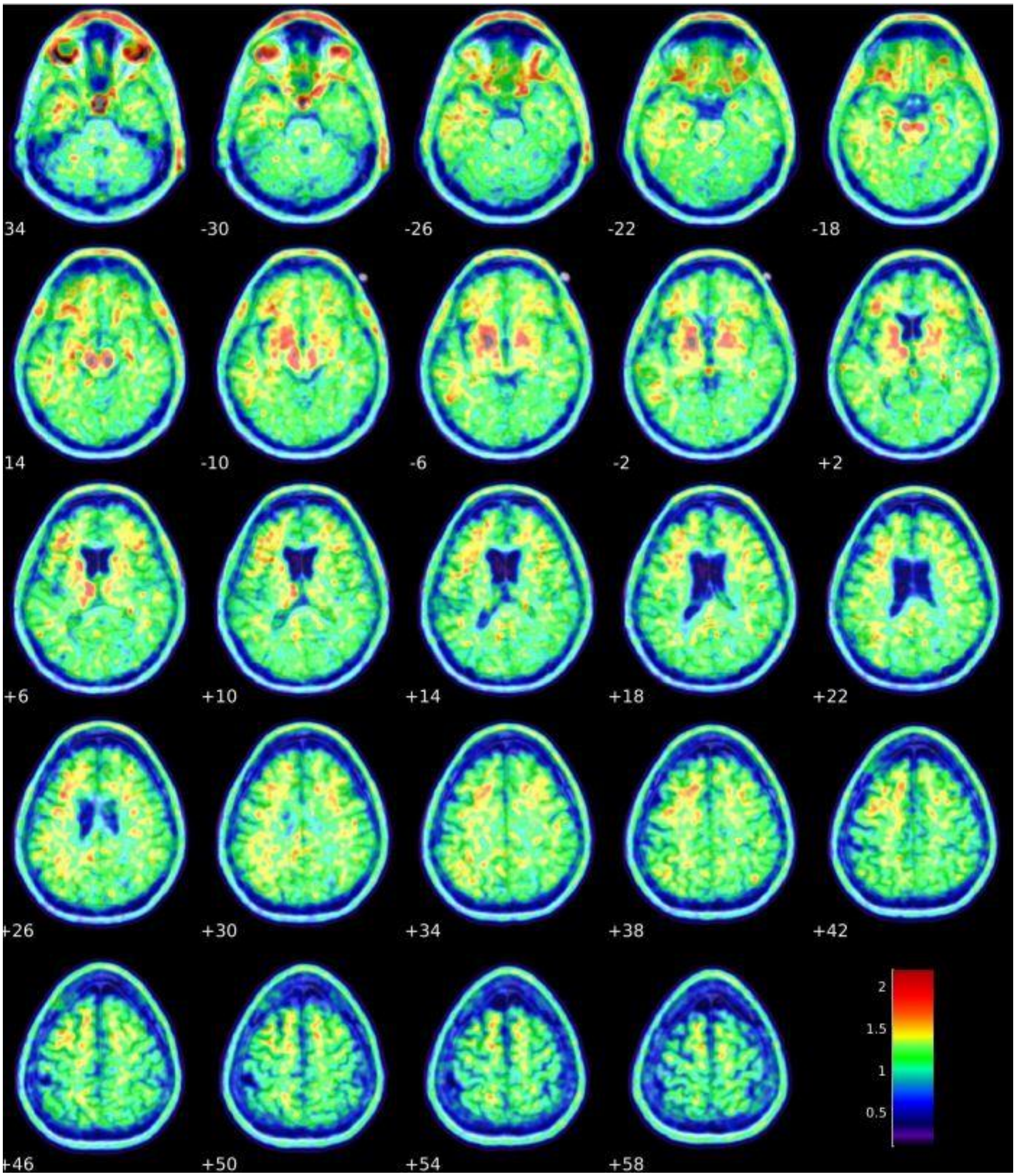

Supplement: Supplementary file 1 — Additional file 1. [file 40478_2021_1197_MOESM1_ESM.pdf]
